# Malignant Melanoma With Neural Marker-Positive Distant Organ Cancers

**DOI:** 10.7759/cureus.65594

**Published:** 2024-07-28

**Authors:** Natsumi Hara, Hitomi Sugino, Natsuko Saito-Sasaki, Etsuko Okada, Yu Sawada

**Affiliations:** 1 Dermatology, University of Occupational and Environmental Health, Kitakyushu, JPN

**Keywords:** case report, immunostaining, histology, tumor marker, malignant melanoma

## Abstract

Malignant melanoma is a melanocyte-derived tumor known for its aggressive clinical behavior. Melanocytes originate from the neural crest, which also gives rise to neural tissues. Malignant melanoma can occasionally exhibit neural differentiation. We report a case of a 70-year-old male with malignant melanoma exhibiting neural marker positivity in the absence of typical melanoma markers. The patient initially presented with a dark nodule on his left heel, which was confirmed as malignant melanoma through cytology. Surgical resection and lymph node dissection were performed, revealing atypical melanocytes. Despite postoperative nivolumab treatment, metastases in the brain and lungs were observed. Histological examination of the brain tumor showed neural differentiation markers (thyroid transcription factor 1 (TTF-1), cytokeratin 7 (CK7), AE1/AE3, and epidermal growth factor receptor (EGFR)) with negative melanoma markers. The patient eventually succumbed to the disease despite multiple treatments. An autopsy revealed multiple organ tumors (brain, duodenum, stomach, liver, and bile duct) negative for melanoma markers but positive for neuroendocrine markers (CD56, synaptophysin, and chromogranin A). This case suggests two possibilities: the coexistence of malignant melanoma with neuroendocrine tumors or a transformation of melanoma into a neuroendocrine phenotype. This case highlights the need for clinicians to consider the potential for melanoma to lose typical markers and transform into neuroendocrine cancer.

## Introduction

Malignant melanoma is a melanocyte-derived malignant tumor known for its aggressive clinical behavior and high metastatic potential [1]. Melanocytes originate from the neural crest, a multipotent structure that also gives rise to various neural tissue components such as spinal and cranial nerves [2]. This shared origin underlies the ability of melanoma cells to exhibit neural differentiation, a phenomenon supported by several previous studies [3,4]. For instance, it has been observed that some melanoma cases express neural markers, reflecting this lineage relationship [4]. Understanding the complex biological behavior of malignant melanoma, including its propensity for neural differentiation, is crucial for accurate diagnosis and effective treatment strategies [5].

In this report, we present a unique case of malignant melanoma with multiple organ cancers showing positive neural markers in the absence of typical melanoma markers. This case underscores the importance of considering neural differentiation in the diagnostic evaluation of melanoma, particularly when traditional markers are absent. In fact, we also encountered significant challenges in diagnosing the case, and it was only through the final autopsy that we confirmed a diagnosis of malignant melanoma. Therefore, enhancing our understanding of the changes in malignant melanoma markers is crucial for informing subsequent treatment.

## Case presentation

**Figure 1:**
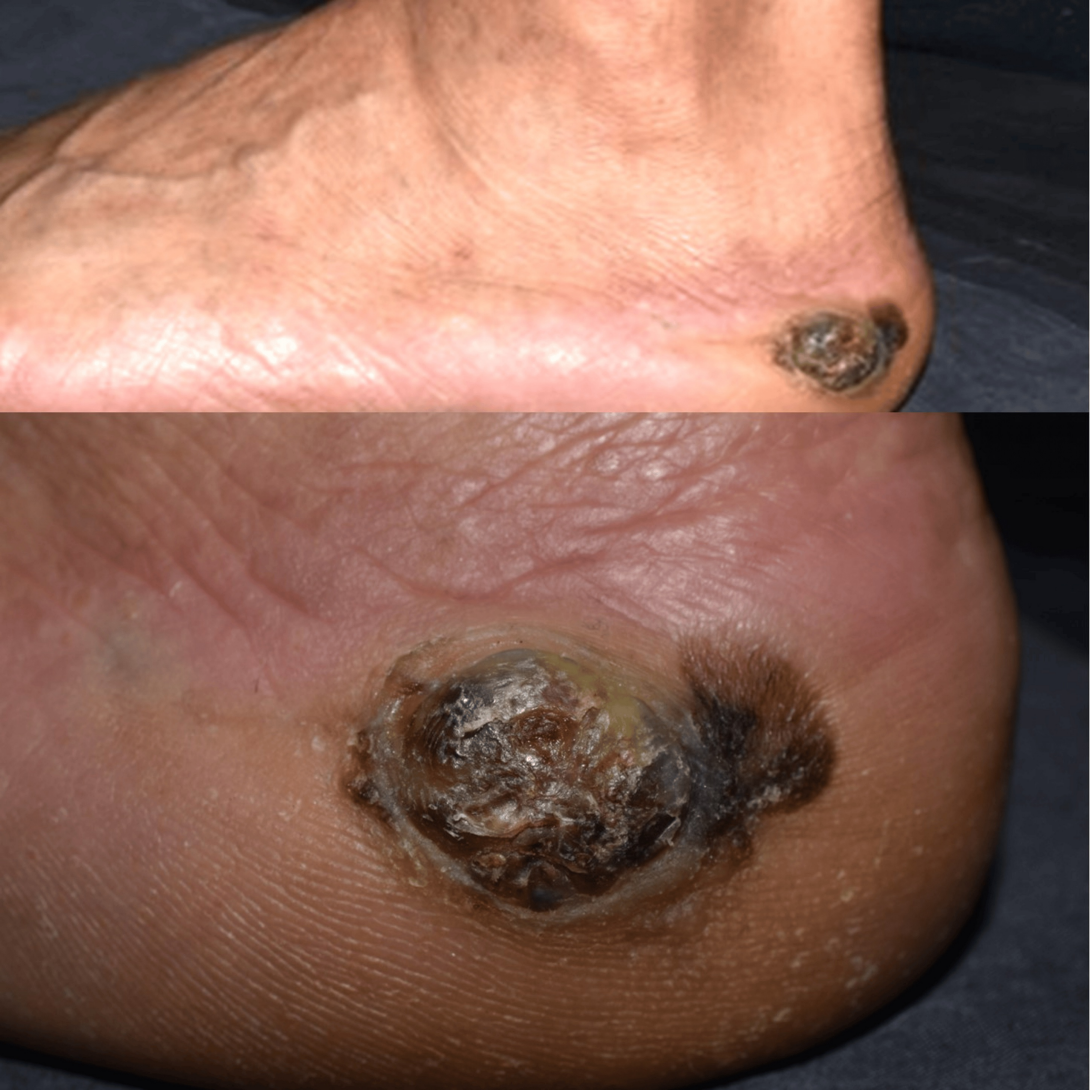
Clinical manifestation of the tumor. A dark tumor measuring 29 x 18 mm in diameter was found on the left heel.

**Figure 2:**
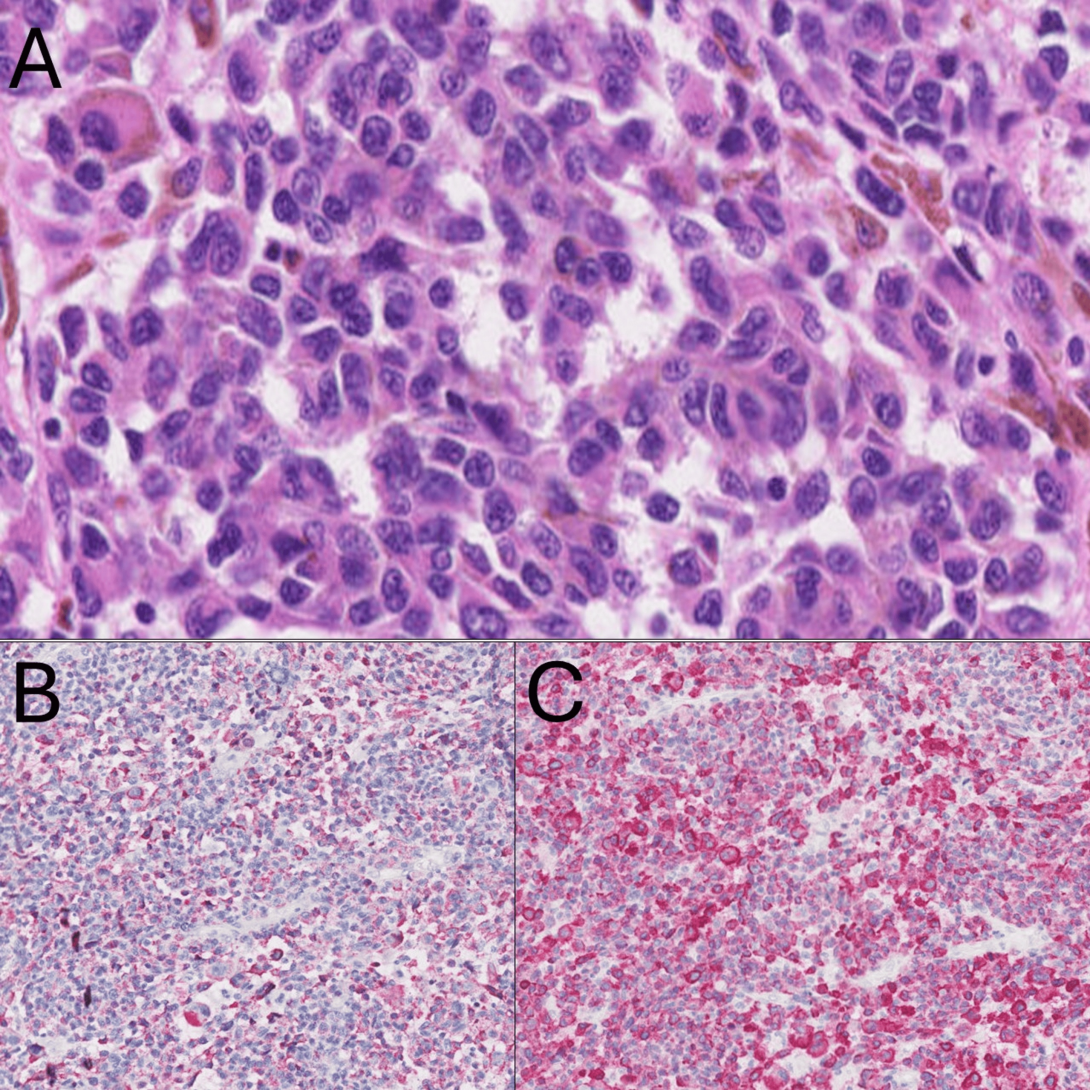
Histological examination. (A) Histological examination of the tumor revealed that atypical melanocytes proliferated larger nuclei in the epidermis and infiltrated into the dermis. (B and C) Immunohistochemical staining showed that HMB-45 (B) and melan-A (C) were positive for tumor cells.

A 70-year-old male noticed a dark nodule on his left heel (Figure 1), which gradually developed for five years. A cytology sample taken from the nodule identified the diagnosis of malignant melanoma. The surgical resection of the tumor and left inguinal lymph node dissection were performed. Histological examination of the tumor revealed that atypical melanocytes proliferated larger nuclei in the epidermis and infiltrated into the dermis (Figure 2). Immunohistochemical staining showed that melan-A and HMB-45 were positive for tumor cells. The left inguinal lymph node metastasis was also identified. No BRAF gene mutation was detected. Based on these examinations, we diagnosed malignant melanoma with pT4bN2cM0 stage IIID.

Nivolumab was initially started as a postoperative adjuvant treatment. We noticed gradually spreading tumors in systemic organs of the brain and lung during the treatment; however, lung tumor was diminished following continuous nivolumab administration. The treatment was discontinued due to immune-related adverse event colitis. Because the brain tumor was specially developed, the tumor was surgically resected. A histological examination of the brain tumor showed that the tumor cells exhibited large-size nuclear mimicking as the primary malignant melanoma metastasis; however, this tumor showed that melanoma markers were completely negative, while tumor cells were positive for thyroid transcription factor 1 (TTF-1), cytokeratin 7 (CK7), AE1/AE3, and epidermal growth factor receptor (EGFR), suggesting a possibility of another malignant tumor, such as lung adenocarcinoma. Although there was a possibility of a transformation into melanoma marker-negative melanoma, the results of completely negative melanoma markers led us to make another possible diagnosis of completely different cancer metastasis. Especially, the brain tumor was a TTF-1-positive tumor, suggesting the cancer originated from a representative TTF-1-positive tumor, such as lung adenocarcinoma metastasis. However, tumor markers of lung adenocarcinoma showed negative results in the sera. Therefore, he received radiation therapy and several forms of chemotherapy, including Taxotere and cyclophosphamide (TC) chemotherapy, as a diagnosis of the metastasis of melanoma without melanoma markers, which were not effective for his tumors. Unfortunately, he died of the underlying tumors.

**Figure 3:**
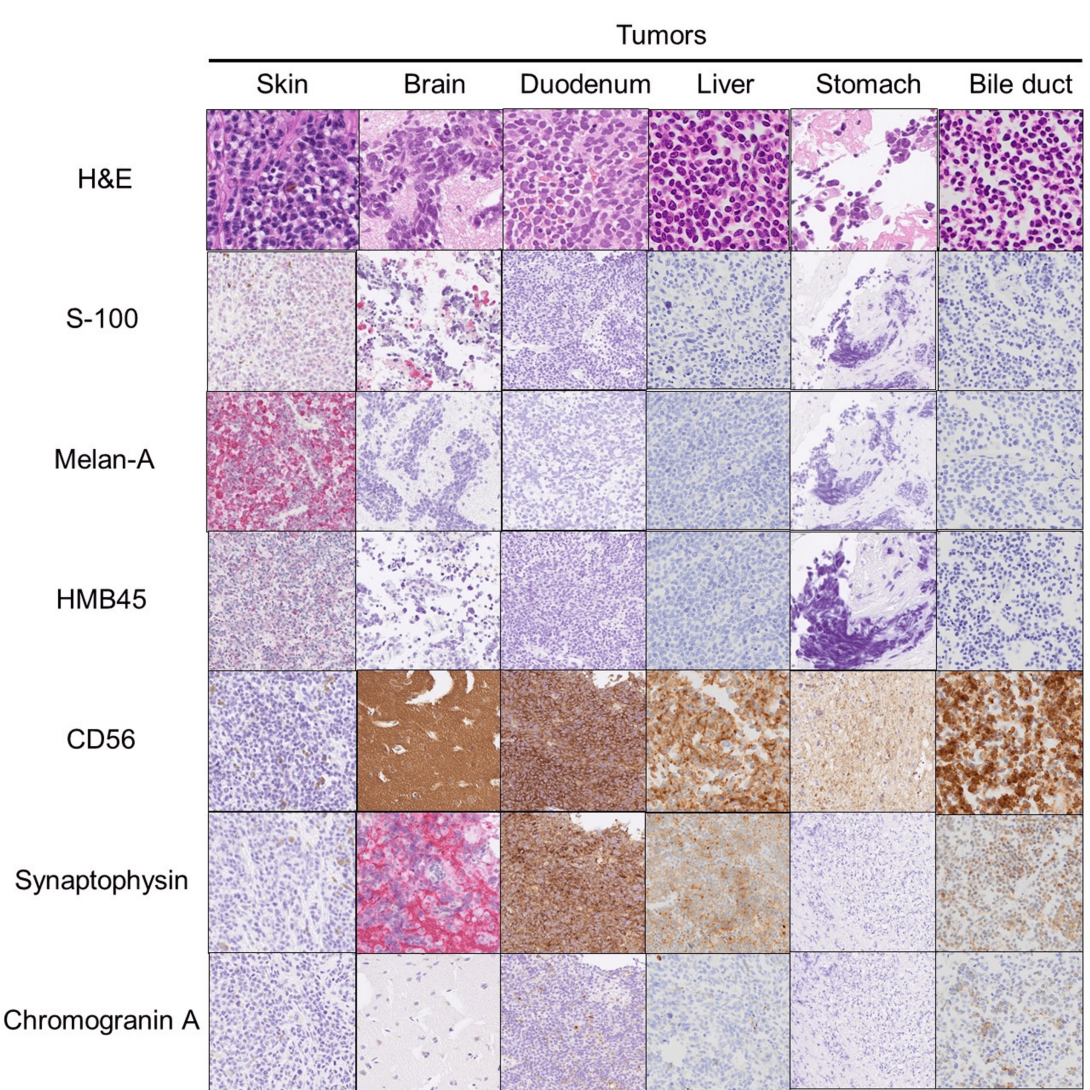
Histological examination of tumors. Histological examinations by hematoxylin & eosin (H&E), S-100, melan-A, HMB45, CD56, synaptophysin, and chromogranin A were displayed.

To clarify the characteristics of multiple organ tumors, we conducted an autopsy-based careful macroscopic examination, which revealed multiple tumors in the brain, duodenum, stomach, liver, and bile duct. Histological examination by hematoxylin & eosin (H&E) staining showed large sizes of the nuclei with round shapes, mimicking the primary malignant melanoma metastasis tumors. However, these tumors were also completely negative for melanoma markers, S-100, melan-A, and HMB-45, but positive for neuroendocrine tumor markers, CD56, synaptophysin, and chromogranin A (Figure 3). These findings indicate that this case exhibited a primary malignant melanoma in the skin and concomitant with neuroendocrine marker-positive malignant tumors or a possible transformation of neuroendocrine tumors from primary malignant melanoma.

## Discussion

This case demonstrated that various sites of multiple tumors were negative for melanoma markers but positive for neuroendocrine markers, suggesting two possible scenarios for the development of these tumors. The first possibility is the accidental occurrence of two entirely distinct malignant tumors: malignant melanoma and neuroendocrine cancers. The second possibility is the transformation of malignant melanoma into neuroendocrine tumors, as previously reported. Malignant melanoma is known to exhibit a variety of histopathological forms, and aberrant expression of immunohistochemical markers can occasionally occur [6]. Additionally, metastatic melanoma has the potential to alter its histological appearance, lose the expression of typical malignant melanoma markers, and mimic other tumors [7].

Interestingly, a retrospective analysis of neuroendocrine marker expression in 308 malignant melanoma cases revealed that 37.2% of cases expressed at least one neuroendocrine marker. Among these, only one case lost all melanoma markers, similar to our case [7]. This finding indicates that malignant melanoma might have the potential to lose its typical markers and transform into a neuroendocrine cancer phenotype in metastatic cases. Clinicians should be aware of the possibility of metastatic melanoma transforming into neuroendocrine cancer even when melanoma markers are completely absent. Moreover, one study conducted an extensive study on the phenotypic plasticity of melanoma, documenting several cases where melanoma cells exhibited a loss of traditional melanocytic markers and acquired alternative marker profiles [8]. Similarly, studies have shown that metastatic melanoma can undergo significant changes in its marker expression profile. For example, another research discusses the clinical implications of melanoma marker variability and the potential for marker loss during metastasis, highlighting the need for comprehensive diagnostic approaches [9].

Furthermore, integrating additional studies exploring the frequency and implications of cellular plasticity in melanoma provides a more robust framework for understanding our case [10]. This study demonstrated that melanoma cells exhibit significant plasticity, enabling them to switch between proliferative and invasive states. This plasticity is driven by changes in the tumor microenvironment and regulatory networks. This concept of cellular plasticity is directly relevant to our findings of melanoma cells exhibiting neuroendocrine differentiation. The ability of melanoma cells to lose traditional melanocytic markers and acquire neuroendocrine characteristics may be driven by similar mechanisms of phenotypic plasticity and microenvironmental influences. This transformation highlights the complexity of melanoma behavior and the need for thorough differential diagnosis using a broad panel of markers to accurately identify and treat these tumors.

Based on these studies, several key points and considerations emerge: the first one is the phenotypic plasticity of malignant melanoma. This plasticity can lead to the acquisition of non-typical marker profiles, complicating diagnosis and treatment. The second one is the loss of melanoma markers. The observation that some melanoma cases may lose their traditional melanoma markers entirely and transform into a neuroendocrine cancer phenotype is crucial. This transformation indicates that melanoma can evolve during metastasis, potentially escaping detection if solely based on melanoma markers. The third one is the clinical implications of these findings. Clinicians should be vigilant about the potential for metastatic melanoma to transform and lose its conventional markers. This awareness is essential for accurate diagnosis and effective treatment planning, as relying solely on traditional markers might lead to misdiagnosis or underdiagnosis. The fourth one is the need for comprehensive diagnostic approaches. Given the variability in marker expression, it is important to employ comprehensive diagnostic methods that go beyond standard melanoma markers. This approach can help detect cases where melanoma cells have undergone phenotypic changes, ensuring accurate identification and appropriate therapeutic strategies. These findings highlight the complex nature of malignant melanoma and the necessity for multifaceted diagnostic approaches to account for potential changes in marker expression during metastasis.

## Conclusions

In summary, this case highlights the rare and complex nature of malignant melanoma, particularly its potential for neural differentiation and transformation into neuroendocrine tumor phenotypes. The patient exhibited a primary malignant melanoma with multiple organ metastases, where the metastatic tumors were positive for neuroendocrine markers and negative for melanoma markers. This phenomenon presents two possibilities: the coexistence of distinct malignant tumors (melanoma and neuroendocrine tumors) or the transformation of melanoma into neuroendocrine tumors. The case underscores the importance of considering such transformations in clinical practice, especially when typical melanoma markers are absent. Our findings align with previous studies suggesting that metastatic melanoma can alter its histological appearance and marker expression, emphasizing the need for comprehensive diagnostic approaches to accurately identify and treat such complex cases.
